# Concordance of Freehand 3D Ultrasound Muscle Measurements With Sarcopenia Parameters in a Geriatric Rehabilitation Ward

**DOI:** 10.1002/jcsm.13648

**Published:** 2024-11-22

**Authors:** Jeremie Huet, Antoine Nordez, Aurélie Sarcher, Marie Mathieu, Christophe Cornu, Anne‐Sophie Boureau

**Affiliations:** ^1^ Nantes Université, CHU Nantes, Movement ‐ Interactions ‐ Performance, MIP, UR 4334 Nantes France; ^2^ Nantes Université, CHU Nantes, Pole de Gérontologie Clinique Nantes France; ^3^ Institut Universitaire de France (IUF) Paris France; ^4^ Nantes Université, CHU Nantes, CNRS, INSERM, l'institut du Thorax Nantes France

**Keywords:** appendicular skeletal muscle mass, elderly, freehand 3D ultrasound, muscle, rehabilitation ward

## Abstract

**Background:**

Sarcopenia is a devastating disease for older adults, but it lacks accessible and reliable tools for measuring total appendicular skeletal muscle mass (ASMM). Two‐dimensional muscle ultrasound (US) has been developed for its bedside clinical advantages and feasibility but lacks standardization and prediction performance. We previously validated a new 3D‐US technique to measure muscle volume (MV) at bedside and applied it in a geriatric rehabilitation setting.

Objectives were to analyse the concordance between 3D‐US MV and ASMM and compare concordance between 3D‐US MV and 2D‐US parameters with ASMM.

**Methods:**

Participants were recruited in a Geriatric rehabilitation ward in Nantes, France, from May to October 2022. Exclusion criteria were as follows: oedema in the lower limbs or recent history of unilateral lower limb damage or stroke. ASMM was measured with bioelectrical impedance analysis; 3D‐US and 2D‐US acquisitions were performed on three muscles of the right lower limb. Measures of strength (hand grip, knee extension and ankle dorsiflexion) were also recorded. Reliability of 3D‐US MV measurements on 10 participants was high (ICC = 0.99).

We used Lin's concordance correlation coefficients (CCC) and bias correction factor for agreement between variables and linear regression models for prediction equations.

**Results:**

Fifty‐eight participants had an interpretable ASMM of whom 17 (29%) had a diagnosis of sarcopenia. Volumes of TA, RF and VL were all significantly concordant with ASMM measured by BIA (all *p* values < 0.001), with CCCs respectively of 0.72, 0.61 and 0.60. MV were all significantly concordant with isometric strength (*p* values < 0.001). Concordance and correlation with ASMM were higher with 3D‐US than 2D‐US measurements regardless of the muscle. Prediction of ASMM reached an adjusted *R* ^2^ of 0.8 with tibialis anterior volume, biometrics and 2D measurements.

**Conclusions:**

This study was the first to use 3D‐US in a geriatric setting and develop a model to predict ASMM in very old hospitalized patients. MV measurements with 3D‐US proved to be reliable and more concordant with appendicular muscle mass and strength than 2D parameters.

## 1 Introduction

Sarcopenia is defined by a progressive and generalized decrease in muscle mass, strength and function. This frequent disease is often devastating, leading to an increased risk of subsequent falls, loss of quality of life, future physical disability, hospitalization, increased health care costs and death [1]. Early detection and intervention are crucial to reduce poor outcomes associated with sarcopenia. Accessible and precise tools for diagnosis in a clinical setting are therefore required [2].

According to the 2019 European Guidelines [1], diagnosis of sarcopenia requires a measurement of muscle quantity and/or quality. Dual‐energy X‐ray absorptiometry (DXA) [3] and bioelectrical impedance analysis (BIA) are well validated for the estimation of total appendicular skeletal muscle mass (ASMM) [4, 5]. However, DXA is irradiating and poorly accessible in everyday care. In addition, both DXA and BIA can only give an overall muscle mass estimation, which could be biased by hydration and fluid distribution [6].

Ultrasound has gained interest in sarcopenia diagnosis for the geriatric community. In addition to being an openly accessible tool in a bedside situation, the multiple innovations still emerging with this technique open up a world of possibilities. Currently, 2D ultrasound (2D‐US) gives access to parameters such as muscle thickness (MT), cross‐sectional area (CSA), pennation angle and fascicle length [7]. These measurements alone have poor predictive values for ASMM [8, 9] and are not validated for the follow up. In addition, standardization of measurements is complex and suffers from the multiplicity of parameters [10].

Muscle volume (MV) is an important outcome to assess muscle hypertrophy [11, 12] or atrophy [13, 14]. Chen et al. have estimated MV with 2D‐US in a geriatric setting indirectly by measuring 5 CSAs on the same muscle [15]. It was better correlated with strength and muscle mass than 2D parameters. However, MV is better measured using freehand 3D ultrasound (3D‐US), and this technique was validated using MRI (i.e., the gold standard) [16, 17]. MV measured by 3D‐US has mostly been used with children with cerebral palsy or young volunteers [18, 19, 20], but never in a geriatric setting. We recently validated with young adults a freehand 3D‐US technique, applicable in a clinical setting with older patients, to measure MV [17, 21].

This study aimed to (i) analyse concordance between 3D‐US MV and ASMM and (ii) compare concordance between 3D‐US MV and 2D‐US parameters with ASMM.

## 2 Methods

### 2.1 Study Population

The DIASEM trial (ClinicalTrials.gov identifier: NCT04753450) was a prospective monocentric study conducted between 1st May and 1st October 2022, which included patients hospitalized at the geriatric rehabilitation ward of the Nantes University Hospital, France. Participants were recruited if their medical condition was stable and if they could walk at least 10 m. Exclusion criteria were: unable to stay still during 3D‐US acquisitions, oedema in the lower limbs, with a recent history of unilateral lower limb damage or stroke (< 3 months) or under judiciary protection.

### 2.2 Ethics

The study was conducted in accordance with the ethical standards set forth in the Declaration of Helsinki (1983). A mandatory French Ethics Committee approved the study design (CPP Ouest III, RC 20_0534). Prior to any data collection, patients were informed of the study and investigator ensured of their informed oral consent specified in the clinical files, as it was approved by the Ethics Committee.

### 2.3 Clinical Assessments

A geriatric assessment was done as it is done in usual practice in a geriatric ward. Clinical data including Mini‐Nutritional Assessment and biometric data were collected for each subject.

ASMM was estimated using BIA (Bodystat QuadScan 4000, QuadScan 4000, Bodystate Ltd, British Islands) and Sergi's equation [1, 5] for all participants. For patients with an indication of bone mineral density measurement, DXA was also performed (LINAR iDXA® with enCORE™software version 12.x).

Skeletal muscle strength was assessed by grip strength, chair stand test and maximum voluntary isometric contractions (MVIC). Hand grip strength was measured using a calibrated Jamar dynamometer (Patterson Medical, Ltd., Nottinghamshire, UK). The participants were instructed to squeeze the dynamometer as hard as possible in a sitting position with the elbow flexed at 90°. Three trials were performed with the dominant hand while the examiner encouraged the participant. Maximum value was kept for analysis. A chair stand test was also performed with five rises, and if impossible, participants could use their hands to help themselves. Time was measured from the “start” instruction of the examiner to the last seating position. Maximum voluntary isometric contractions of the right lower limb (i.e., knee extensors and ankle dorsiflexors strength assessments) were performed. We used the microFET®2 hand‐held dynamometer (Hoggan Scientific, LLC) as previously validated in older adults [22]. For knee extensor, participants were sitting at the edge of the bed with their leg hanging at a 90° angle, whereas the examiner held firmly the dynamometer approximately 5 cm above the ankle. Participants were instructed to extend their lower limb as hard as they could without touching the bed with their arms, whereas the examiner was encouraging them. Each measurement was done three times with a minimal 2‐min break in‐between, and the maximum peak was recorded. Distance between the dynamometer and the centre of the knee was measured to calculate torque. For ankle dorsiflexion, the participant was kept in a supine position with the ankle at 90°, and the dynamometer was positioned between the first and second metatarsophalangeal joint. The maximum peak of three measurements was kept for analysis.

Physical performance was assessed by gait speed, measured in meters per second using a 6‐m walking test.

### 2.4 Ultrasound Measurements

An Aixplorer US scanner (version 11.0, Supersonic Imagine, Aix‐en‐Provence, France) was used for B‐mode US acquisitions, with a linear transducer (4–15 MHz, Superlinear 15‐4, Vermon, Tours, France).

**FIGURE 1:**
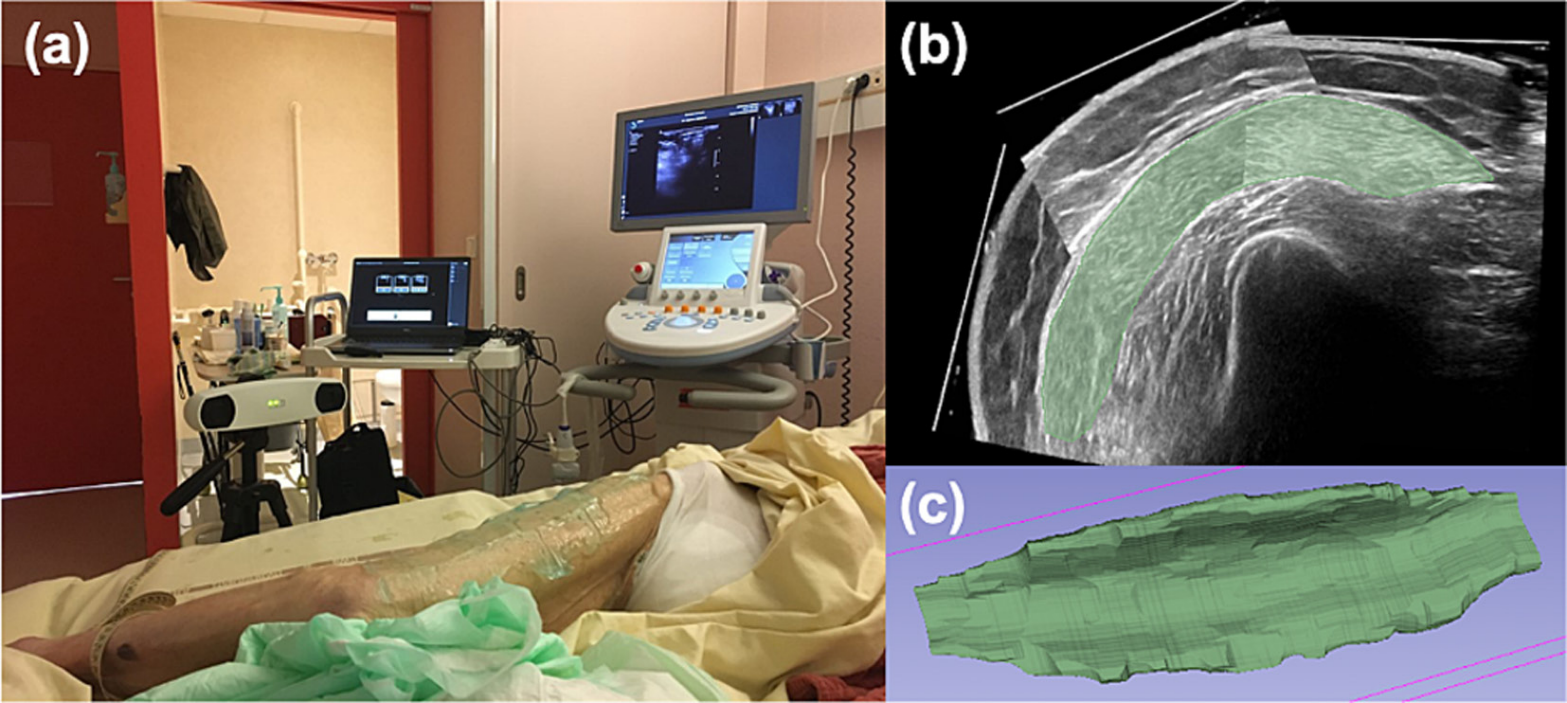
Visualization of the 3D‐US setup and muscle segmentation. (a) Bedside setup in a hospital ward for a vastus lateralis scanning. (b) Example of 1 slice in axial plane of the vastus lateralis with module “segment editor” of 3D Slicer. (c) Three‐dimensional visualization of completed segmentation in 3D Slicer.

For 3D‐US MV measurements, our method was previously validated in young healthy participants and thoroughly described in a previous article [21]. Briefly, a copious amount of gel and a trained examiner (over 100 h of acquisitions) allowed to minimize muscle compression, ensuring that measurements were not biased. For spatial tracking, we used a double optical camera (NDI, model Polaris Vicra Position Sensor, May 2013, Canada) to follow optical markers attached to the probe. A PiurImaging software (Piur tUS, PiurImaging GmbH, Vienna, Austria) installed on a separate laptop performed an automatic and instantaneous volume reconstruction. Data were then exported to DICOM format. We used 3D‐Slicer for the segmentation process in order to calculate the volume of each muscle (Figure 1).

For 2D‐US measurements, following Perkisas' standardization, we extracted a medial CSA at 50% of length for each muscle, orthogonal to its axis, in order to measure CSA area and MT [10]. These measurements were performed without the 3D system.

Three muscles of the right lower limb per participant were scanned: the tibialis anterior (TA, lying supine), the rectus femoris (RF, lying supine) and the vastus lateralis (VL, lying on the left side). For decency reasons with its proximal insertion, scans of RF were stopped proximally on the inguinal fold using a mark on the skin (perpendicular to the leg axis). Scans of TA were stopped distally just above the external malleolus.

### 2.5 Reliability of Freehand 3D‐US MV Measurements in a Geriatric Setting

As previously reported, freehand 3D‐US has never been used in a geriatric setting to measure MV. Moreover, the method used, in particular to correct the probe's compression effect on MV estimation, is new and only validated on young healthy participants [21]. Therefore, for 10 volunteers among included participants, volume measurements were repeated a second time in order to test reliability. This second acquisition was performed under 3 days of the first. The two‐way random intraclass correlation coefficients (ICCs) was 0.99 (CI = [0.99; 1]). Coefficient of variation (CV) and standard error of measurement (SEM) were respectively 3.9% and 6 mL. For the TA, RF and VL, ICCs were 0.97 (CI = [0.91; 0.99], CV = 4.2%, SEM = 7 mL), 0.98 (CI = [0.94; 0.99], CV = 3.8%, SEM = 4 mL) and 0.99 (CI = [0.96; 1], CV = 3%, SEM = 6 mL), respectively.

### 2.6 Reliability of Hand‐Held Dynamometer for Measuring MVIC

MVICs were also repeated a second time to assess reliability. The two‐way random ICCs were respectively 0.96 (CI = [0.89; 0.99]) for knee extension and 0.85 (CI = [0.52; 0.95]) for ankle dorsiflexion. CV were respectively 8.6% and 9.5%. SEM were respectively 4.5 N m and 14 N.

To compare, ICC for handgrip was 0.99 (CI = [0.98; 1]), CV 2% and SEM 4 N.

### 2.7 Statistical Analysis

Grubbs' test was used to exclude outliers (BIA measurements inconsistent results with the participant's biometric properties) from the analysis, after verification of the normal distribution with a Q‐Q plot.

Descriptive statistics for continuous variables were expressed as means and standard deviations for Gaussian variables or median and interquartile range for non‐Gaussian variables. Non‐Gaussian was determined graphically and with a Shapiro test. Categorical variables are presented as numbers (*n*) and percentages.

To measure concordance, variables were first standardized as *Z* score for Gaussian variables or scaled as a percentile of range for non‐Gaussian variables. Lin's concordance correlation coefficients (CCC) and bias correction factors (Cb) were used to measure concordance between variables (volume, CSA, MT, measures of strength and physical performance). Moreover, Lin's concordance coefficient = Pearson's correlation × Cb. A gender‐specific analysis was also performed to evaluate discrepancies between genders. Bland–Altman plots were generated to analyse concordance graphically between ASMM and MV. Pearson's correlation coefficients were also calculated and summarized in a heatmap to extract clusters of correlation.

Multivariate linear regression analysis was used to build a model to predict ASMM with biometric data and MVs. Power of the model was calculated using adjusted *R* ^2^. Minimal effective models were determined by minimization of Aikake's information criterion [23] or Lasso method [24]. All *p* values were two sided, and *p* values less than 0.05 were considered statistically significant.

All statistics were performed using the R statistical package, version 3.6.2 (R Foundation for Statistical Computing, Vienna, Austria).

## 3 Results

### 3.1 Patient Characteristics

The mean age of the 60 included participants was 86 years old, and 22 were men (36.7%). Two results of BIA were considered as outliers and inconsistent with other biometrics, thus excluded from the analysis. Table 1 summarizes the characteristics of the 58 analysed patients. Among them, 17 (29.3%) had a diagnosis of sarcopenia according to the 2019 European consensus definition [1].

**TABLE 1:** Patient characteristics.

Variable	*N*	Women (*N* = 38)	Men (*N* = 20)	Overall	*p*
Age (years)^1^	58	86 (± 5)	86 (± 5)	86 (± 5)	0.95
Height (cm)^1^	58	158 (± 7)	167 (± 4)	161 (± 8)	**< 0.001**
Body mass (kg)^1^	58	56 (± 10)	70 (± 11)	61 (± 12)	**< 0.001**
BMI (kg/m^2^)^1^	58	23 (± 4)	25 (± 4)	23 (± 4)	**0.03**
ASMM (kg)^1^	58	14 (± 3)	20 (± 3)	16 (± 4)	**< 0.001**
Hand grip (kg)^2^	58	14 (13–16)	24 (19–28)	16 (14–22)	**< 0.001**
Knee extension torque (N m)^1^	56	39 (± 14)	61 (± 20)	47 (± 20)	**< 0.001**
MVIC dorsiflexion (kg)^1^	54	15 (± 4)	18 (± 5)	16 (± 4)	**0.01**
Gait speed (m/s)^1^	52	0.7 (± 0.3)	0.7 (± 0.4)	0.7 (± 0.3)	0.8
TA volume (mL)^1^	56	141 (± 26)	185 (± 30)	156 (± 34)	**< 0.001**
RF volume (mL)^2^	58	86 (73–97)	122 (111–141)	92 (79–113)	**< 0.001**
VL volume (mL)^2^	56	176 (146–209)	264 (220–328)	203 (160–241)	**< 0.001**

*Note:* Values are expressed as ^1^mean (sd) if Gaussian or ^2^median (IQR) if non‐Gaussian. *p* values < 0.05 are presented in bold.

Abbreviations: ASMM, total appendicular skeletal muscle mass; BMI, body mass index; TA, tibialis anterior; RF, rectus femoris; VL, vastus lateralis.

As expected, there was a significant difference between men and women in terms of biometrics, strength, ASMM or MVs (all *p* values < 0.001). Mean body mass was 56 kg (± 10) for women and 70 kg (± 11) for men, with mean ASMM respectively of 14 (± 3) and 20 (± 3) kg. Hand grip was significantly different with a mean of 15 kg (13–16) for women and 24 kg (19–28) for men, whereas gait speed was not significantly different with an overall mean of 0.7 m/s (± 0.3) (*p* value = 0.8).

### 3.2 Concordance Between 3D‐US MV and ASMM, Comparison With 2D‐US Parameters

Table 2 shows the concordance matrix between 3D‐US measurements and muscle mass variables. It shows Lin's concordance correlation coefficients and bias correction factors.

**TABLE 2:** Concordance and accuracy matrix of ultrasound measurements with muscle mass and other sarcopenia parameters.

	Tibialis anterior			Rectus femoris			Vastus Lateralis		
	Volume	CSA	MT	Volume	CSA	MT	Volume	CSA	MT
BIA ASMM	0.72 * (1)	0.43 * (1)	0.41 * (1)	0.61 * (0.96)	0.44 * (0.91)	0.26* (0.85)	0.60 * (0.93)	0.40 * (1)	0.25 (1)

DXA ASMM	0.68 * (0.99)	0.56 * (1)	0.41 (1)	0.68 * (0.95)	0.54 * (0.87)	0.37* (0.79)	0.80 * (0.98)	0.64 * (0.98)	0.43 * (0.97)
Grip strength	0.51 * (0.92)	0.18 (0.79)	0.18 (0.78)	0.56 * (0.93)	0.31* (0.98)	0.27* (0.97)	0.51 * (0.98)	0.33 (0.90)	0.19 (0.76)
MVIC dorsiflexion	0.48 * (1)	0.06 (1.00)	0 (1.00)	0.34* (0.90)	0.19 (0.83)	0.14 (0.82)	0.27* (0.82)	0.17 (1)	0.21 (1)
Knee extension torque	0.51 * (1)	0.25 (1)	0.24 (1)	0.48 * (0.98)	0.30* (0.93)	0.22 (0.90)	0.58 * (0.92)	0.53 * (1)	0.42 * (1)

FTSS	−0.03 (0.87)	0.06 (0.73)	−0.04 (0.71)	−0.22 (0.86)	−0.18 (0.89)	−0.09 (0.86)	−0.11 (0.91)	−0.04 (0.82)	0 (0.65)
Gait Speed	0 (1)	−0.26 (1)	−0.08 (1)	0.12 (0.98)	0.18 (0.97)	0.18 (0.90)	0.01 (0.95)	−0.06 (1)	0.10 (1)

*Note:* Values are expressed as Lin's concordance correlation coefficient (precision) and bias correction factor (accuracy) in parenthesis. As variables are not the same unit, they have been previously standardized as *Z* score if Gaussian or scaled as percentile of max–min if one of both is non‐Gaussian. To note, Pearson's correlation coefficient = lin's CCC/bias correction factor. Therefore, if bias correction factor = 1, then Lin's CCC = Pearson's correlation. Darker blue expresses higher concordance correlation coefficients. Nonsignificant coefficients or under < 0.2 are marked white.

Abbreviations: BIA ASMM, appendicular skeletal muscle mass measured with bioelectrical impedance analysis; BMI, body mass index; DXA ASMM, appendicular skeletal muscle mass measured with dual energy X‐ray absorptiometry; FTSS, five times to sit and stand‐up test; MVIC, maximum voluntary isometric contraction.

* *p* value < 0.05.

Volumes of TA, RF and VL were all significantly correlated with ASMM measured by BIA (all *p* values < 0.001), with concordance coefficients (precision) respectively of 0.72, 0.61 and 0.60 and bias correction factors (accuracy) of 1, 0.96 and 0.93. On only 22 subjects with DXA, concordance between 3D‐US MV and DXA ASMM were still highly significant (all *p* values < 0.001) and with estimated CCCs of 0.68, 0.68 and 0.80 for TA, RF and VL, respectively (Table 2).

**FIGURE 2:**
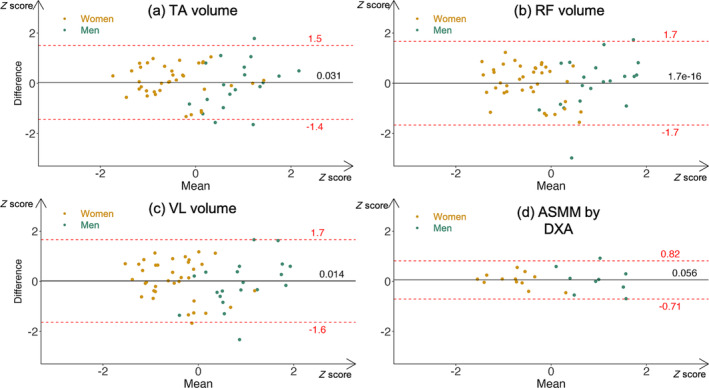
Bland–Altman plots of BIA ASMM versus freehand 3D‐US Muscle Volumes and DXA ASMM. Variables were previously standardized with a Z‐score to allow for comparison. Women (gold) and men (green) are distinguished with colours. Black continuous lines represent mean biases. Dashed red lines represent 95% limits of agreement. To note, all Bradley–Blackwood *F* tests are nonsignificant, implying concordance. ASMM: appendicular skeletal muscle mass; BIA: bioelectrical impedance analysis; DXA: dual x‐ray absorptiometry; TA: tibialis anterior; RF: rectus femoris; VL: vastus lateralis.

Figure 2 shows Bland–Altman plots comparing each MV and DXA ASMM with BIA ASMM. Mean biases are all inferior to 0.1 *Z* score, and limits of agreement are all under 2 *Z* scores, which shows good agreement between measurements. Compared with DXA, limits of agreement are approximately twice as far but distribution shows the same normality around the *Y*‐axis with a sex‐specific distribution around the *X*‐axis.

**FIGURE 3:**
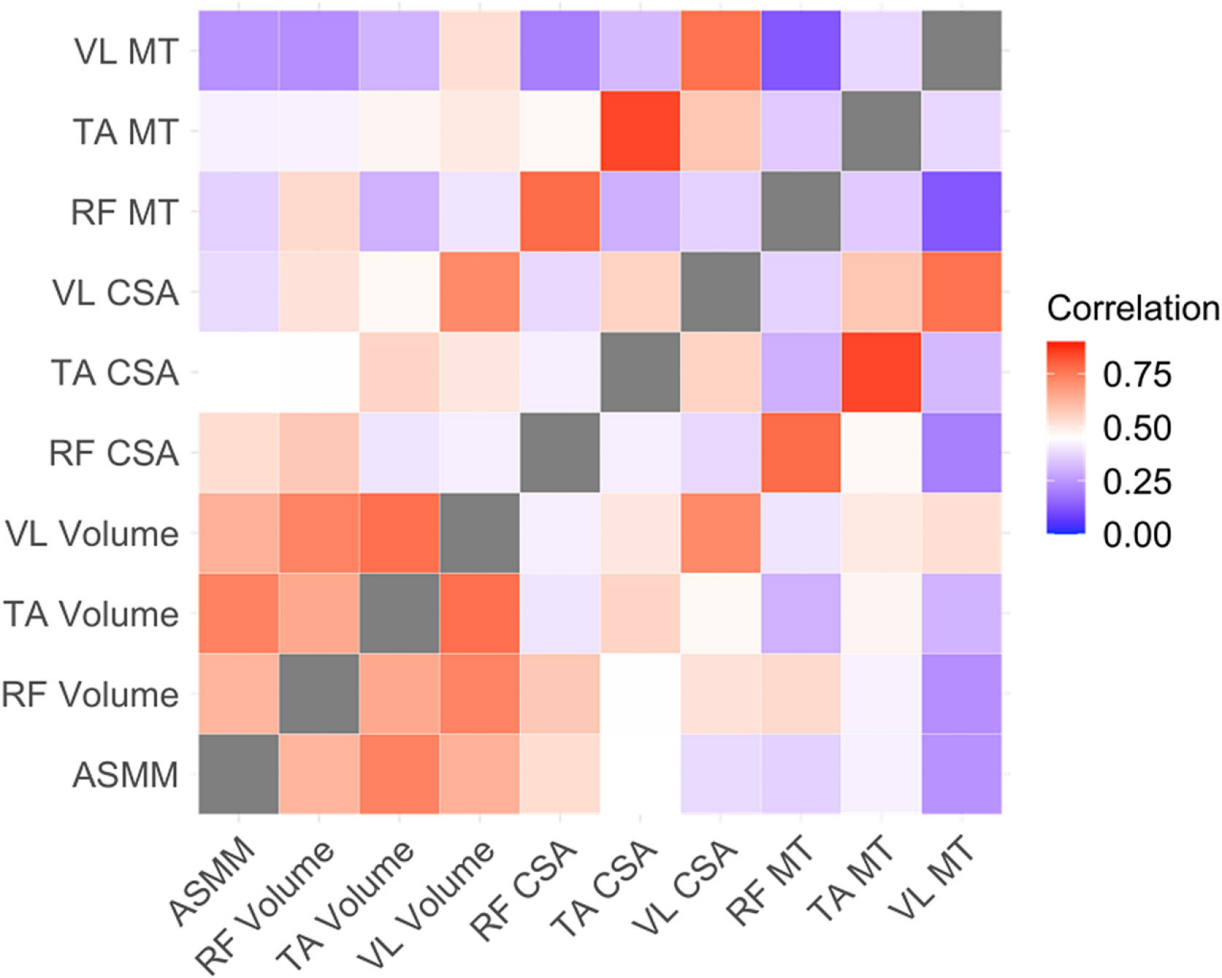
Heatmap of correlation matrix with ASMM and US muscle measurements. Dark red shows high correlation coefficients, whereas dark blue shows low correlation coefficients. White is midpoint. ASMM: appendicular skeletal muscle mass; RF: rectus femoris; TA: tibialis anterior; VL: vastus lateralis; CSA: cross sectional area; MT: muscle thickness.

3D‐US MV had higher CCCs with ASMM than 2D‐US parameters (CSA and MT), regardless of the muscle. In addition, our heatmap shows a correlation cluster between ASMM and MVs, leaving aside 2D‐parameters (Figure 3).

### 3.3 Concordance With Other Sarcopenia Parameters

3D‐US MV were also well correlated with measures of strength (Table 2). For example, for TA, grip strength and ankle dorsiflexion were precisely correlated to its volume with respectively CCC = 0.51, *p* value < 0.001 and CCC = 0.48, *p* value < 0.001. These results were better than 2D‐US parameters, as CCCs for cross‐sectional area and muscle thickness with ankle dorsiflexion were respectively 0.06, *p* value = 0.3 and 0, *p* value = 1.

### 3.4 Gender‐Specific Analysis of Concordance and Correlation

**FIGURE 4:**
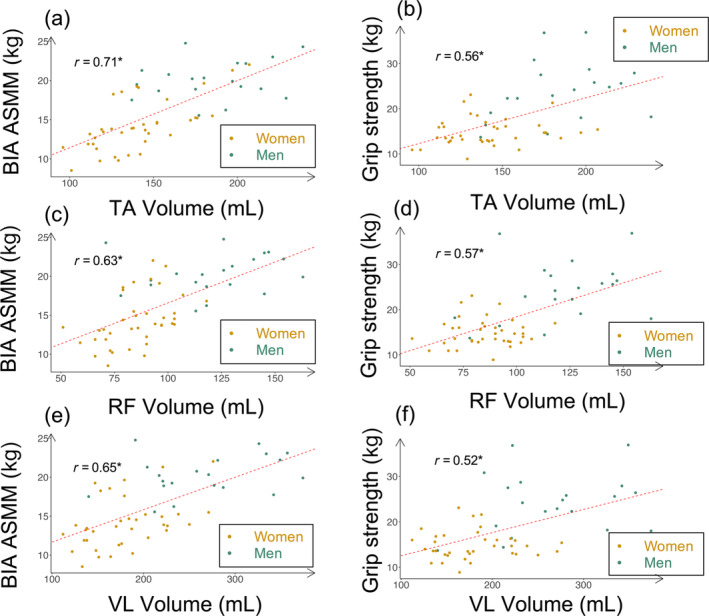
Gender‐colourized correlation plots of freehand 3D‐US muscle volumes with ASMM and grip strength. Subfigures (a), (c) and (e): correlation plots of muscle volumes with ASMM. Subfigures (b), (d) and (f): correlation plots of muscle volumes with grip strength. Men are colourized in green and women in gold. Dashed red lines represent linear regression slope. “*r* = *x*” shows global Pearson's correlation coefficient between variables. To note, all correlations are significant, as the “*” indicates. ASMM: appendicular skeletal muscle mass; BIA: bioelectrical impedance analysis; TA: tibialis anterior; RF: rectus femoris; VL: vastus lateralis.

Tables S1 and S2 in our supporting information show a gender‐specific analysis of the same matrix. Volume concordance with ASMM was significant in women (*n* = 38) but not in men (*n* = 20), in whom concordance with strength was significant. Figure 4a–f shows gender‐colourized correlation graphs of MV with both ASMM and grip strength. Figures S1 and S2 show the same graphs for all ultrasound parameters.

### 3.5 Prediction of ASMM

Models are summarized in Table 3. The initial predictors including biometrics (height, body mass and gender) and the three MV were entered in a multivariate regression model that explained 77% of the variance in ASMM.

**TABLE 3:** Multivariate linear regression models for predicting ASMM (in kg) estimated with BIA.

Model	Coefficient	Adjusted *R* ^2^	RSE	*p*
Biometrics		0.75	2.05	< 0.001*
+ TA volume	0.022			0.06
Biometrics		0.77	1.98	< 0.001*
+ TA volume	0.034			0.01*
+ VL volume	0.012			0.11
Biometrics		0.77	1.99	< 0.001*
+ TA volume	0.034			0.02
+ RF volume	0.012			0.51
+ VL volume	−0.014			0.09
Biometrics		0.80	1.98	< 0.001*
+ TA volume	0.025			0.03*
+ RF CSA	0.64			0.05*
+ VL CSA	−0.25			0.04*
Biometrics		0.77	2.03	< 0.001*
+ TA CSA	0.32			0.14
+ RF CSA	0.51			0.06
+ VL CSA	−0.29			0.04*

*Note:* Models were determined for best fit using Lasso method and minimization of Akaike's information criterion. We chose to present different models in order to compare predictive values with adjusted *R* ^2^. A model with three volumes was also forced because these measurements were of most interest.

Abbreviations: ASMM, appendicular skeletal muscle mass; BIA, bioelectrical impedance analysis; CSA, cross‐sectional area; RSE, residual standard error; TA, tibialis anterior; VL, vastus lateralis; RF, rectus femoris.

* *p* < 0.05.

With only TA volume, multivariate regression model explained 75% of the variance in ASMM.

A linear model including biometrics (body mass, height and gender), TA volume and VL volume were determined as the minimal effective model with only 3D parameters.

By adding 2D parameters to our predictive outcome, our minimal effective model included biometrics, TA volume, RF CSA and VL CSA, with an adjusted *R* ^2^ of 0.80. This model concurred with best fit using the Lasso method. To note, adjusted *R* ^2^ with three CSAs was lower (0.77), and residual standard error was higher.

## 4 Discussion

Lower limb MV measurements performed in a geriatric rehabilitation ward using freehand 3D‐US were reliable and showed good concordance to total ASMM. Estimated concordance correlation coefficients range between 0.60 and 0.80. In addition, volume was better correlated to measures of strength and muscle mass than the previously reported 2D parameters. Lastly, we developed a model to predict total ASMM with MVs with an adjusted *R* ^2^ of 0.80.

### 4.1 Concordance Between MV and ASMM, Comparison With Muscle CSA and Thickness

To our knowledge, freehand 3D‐US has never been previously used in a geriatric setting. However, Chen and colleagues estimated RF volume with five CSAs and an elliptical cylinder method [15]. In their study, the correlation coefficient of RF MV with appendicular skeletal muscle index was 0.86, which is higher than our findings (0.63). However, their participants included community‐dwelling adults with a mean age of 68 years old, versus 86 in our study. Therefore, our range of volumes was thinner (maximum RF 160 vs. 200 mL), reducing artificially our correlation values, but our included population is more representative of a geriatric rehabilitation context.

In addition, compared with MRI, MV measurement is more accurate with 3D‐US [17, 18] than by interpolating multiple CSAs using 2D techniques [16]. Our results show good agreement between methods with Bland–Altman plots (Figure 2), which are not available in this compared article. However, their results show the same trend with a prediction capacity of three‐dimensional > two‐dimensional > one‐dimensional.

For 2D‐US parameters, we obtained CCCs between 0.25 and 0.41 for MT (correlations 0.25–0.43), between 0.40 and 0.64 for CSA (correlations 0.39–0.48). In the same way, these findings are lower than previous studies. Berger et al. measured a correlation coefficient of 0.70 and a Lin's CCC of 0.68 for RF MT in healthy volunteers with a mean age of 73 years old [25]. Abe et al. measured a correlation coefficient of 0.57 in 60‐year‐old Japanese healthy volunteers [26]. But these studies included participants who were healthier, younger, less frail and less morbid than our population. To support this, Seymour et al. compared correlation coefficients between healthy older adults and patients of the same age with chronic obstructive pulmonary disease. They found that correlation coefficients of RF CSA with BIA‐derived fat‐free mass decreased from 0.66 to 0.43 in patients with comorbidities [27]. To our knowledge, no study has included hospitalized patients of a comparable age. Madden included 150 patients of geriatric clinics, but mean age was significantly lower (80 years old) and correlation coefficients between MT and ASMM or univariate regression *R* ^2^ are not available [28].

### 4.2 Prediction Equations of ASMM

Our study was the first to develop a prediction equation using direct measurement of MV. With biometrics, TA volume, RF CSA and VL CSA, our model's adjusted *R* ^2^ was 0.8. Again, Chen's regression model had an adjusted *R* ^2^ of 0.85 to predict muscle mass but used an indirect estimation of MV [15]. Previous researchers have focused mainly on MT to predict total muscle mass with ultrasound. Abe et al. used MT of four sites to predict DXA‐derived total appendicular lean mass and obtained an adjusted *R* ^2^ of 0.90. They included 389 healthy Japanese volunteers with a mean age of 71 years old [8]. Takai et al. included 77 healthy volunteers with a mean age of 61 years old and developed a prediction equation using 4 MT. Their adjusted *R* ^2^ was 0.96 [29]. More recently, Tang et al. validated a muscle mass estimation equation with MT at four sites in a cohort of 669 older adults with a mean age of 71 years old. The ICC between ultrasound‐derived ASMM and BIA‐measured ASMM was 0.885 [30]. Our linear regression model had slightly lower predictive capacities than previous research but included only hospitalized patients of very old age and a high level of comorbidities. Indeed, for 80‐year‐old patients of geriatric clinics, *R* ^2^ for lean body mass reached only 0.58 with biometrics and MT [28]. Other phenomena associated with aging and frailty, such as muscle fat infiltration, can explain this gap [31]. Moreover, studies have shown that sarcopenia is not uniform and may alter preferably quadriceps and abdominal muscles [32]. Furthermore, hospitalized older patients have numerous comorbidities, such as dementia, heart failure, malnutrition and a history of lower limb fracture. As muscle ultrasound is influenced by various conditions affecting muscle quantity and quality (position, fat infiltration, oedema), the correlation results may be reduced by the variability in hydration and intracellular/extracellular fluid balance.

Overall, comparing our results with other studies is difficult, given the population included and the choice of MV through 3D‐US. Therefore, comparing correlation coefficients inside our data seems also important.

### 4.3 Advantages of MV Measurements With 3D‐US

MV showed higher predictive values than 2D parameters in this population. Previous research has extensively validated the reliability of 2D‐US measurements [9], but efforts of standardization have shown the lack of repeatability between studies, in particular on the anatomical choice for measurement [10]. Our experience with 2D measurements confirms the difficulties in position and orientation of the probe for measurements. For example, there is no standardization of 2D‐US measurement of tibialis anterior. Some authors choose a 30% proximal landmark [33], whereas others measure at 50% length with no clear anatomical landmark [34], likewise to other muscles even though its unique form cannot be compared with other belly‐shaped muscles such as the RF. In contrast, MV with freehand 3D‐US is reliable [17, 18, 21], as confirmed in our study (ICC 0.99), and is a unique measurement, thus resistant to repeatability issues.

### 4.4 Concordance With Skeletal Muscle Strength and Physical Performance

Our study also evaluated MVIC of knee extension, ankle dorsiflexion and hand grip using hand‐held dynamometers. Concordance with MVs was significant, with CCCs between 0.27 and 0.58; in contrast, the results for 2D parameters were less straightforward. As expected, the concordance of MVIC with their associated muscular group volumes was significant. But, to note, good concordance was also found between grip strength and MVs. Thomaes and colleagues measured a correlation coefficient of 0.61 between RF MT and MVIC of knee extensors in older outpatients with coronary artery disease [35], and Strasser calculated a correlation coefficient at 0.83 in a physical rehabilitation clinic [36], both with a mean age of 68 years old. In patients with chronic kidney disease, RF CSA was significantly correlated with MVIC (*r* = 0.3) [37]. Thus, estimations are variable between authors and methods used. Moreover, previous results suggest that correlation coefficients decrease with age [36]. In addition to total body fat, muscle fat infiltration also increases with age, which could explain the gap between ultrasound parameters and strength or total lean mass, as suggested by Delmonico and colleagues in 2009 [31].

In our experience, even if previous authors showed comforting results for reliability [22, 38], grip strength was more reproducible between measurements than knee extension and ankle dorsiflexion MVIC, as confirmed by our reliability measurements, probably due to comorbidities, common apathy and sometimes depression, which could cause difficulties to produce maximal strength. Moreover, hand‐held dynamometers depend on the examiner's counterforce, which is most variable in ankle dorsiflexion. This might explain higher ICCs with grip strength (0.99) versus knee extension (0.96) and ankle dorsiflexion (0.85) and reinforce its importance in everyday care as a measurement of strength.

Gait speed was very poorly correlated with other measurements in our study population, as already pointed out in previous studies including older patients in a geriatric ward [28]. This is probably explained by a high prevalence of other confounding factors such as heart/respiratory failure, knee arthritis, fear of falls or balance and gait impairments after falls.

### 4.5 Limitations

Our study has several limitations. First of all, MV measurement with freehand 3D‐US is still a time‐consuming technique compared with MT. It takes approximately 10 min of scanning and 30 min of segmentation per muscle. It is not, for the moment, an easy procedure: it requires a specific training of approximately 30 h for reliable measurements, a dedicated optical tracking system and a powerful computer for volume reconstruction and segmentation. However, our study showed that scanning of the TA alone could approach closely the optimal model with an adjusted *R* ^2^ of 0.75. This could limit data extraction time to 40 min per participant. Moreover, automatic segmentation of MV should be available in a few years, as a lot of progress has been accomplished in this field recently [39].

Secondly, our number of observations was too low to interpret a gender‐specific analysis. Power and range of values were insufficient. Even if we can see differences between men and women (Tables S1 and S2) regarding concordance with ASMM or strength, it is difficult to extract a general trend. Figures 2 and 4 show graphically the gender‐specific distribution of MVs and ASMM. The same distribution appears for strength. In our multivariate analysis, gender was not significant once we added body mass and volume, although Sergi's equation of BIA ASMM depends on gender [5]. Therefore, the differences in strength and ASMM between genders (Table 1) seem to be largely explained by the differences in MV, although MV was calculated regardless of gender. For example, two women had a TA volume above men's median, and we found the exact same patients for ASMM. Therefore, the gender differences observed reinforce the predictive capacity of MV as a gender‐neutral biomarker.

Thirdly, for some participants, MV measurements may be biased by their nonadherence to the examiner's instructions. In our study, only 10% showed a lack of compliance during acquisitions, but participants were preselected. This phenomenon could increase if generalized to all patients. However, the acquisition of TA volume was the least constraining for our participants and compliance decreased with time. This supports the relevance to focus on TA volume measurement in the future.

Fourthly, as stated before, measurements of strength and physical performance could be biased by patients' frailty, apathy or by the examiner himself. ICCs and CV were better with MV than with hand‐held dynamometers. This may reinforce the importance of measuring MV, which could represent an objective measurement of localized muscle mass in patients where strength measurements can be challenging. Therefore, MV could become a more objective tool to follow efficacy of nutritional or exercise interventions in sarcopenia.

Also, ultrasound, DXA and BIA measurements all suffer from changes with hydration status [3], and evaluating hydration is commonly challenging in geriatrics. However, ultrasound is a local evaluation and is precisely the technique of choice in clinical practice for detecting generalized oedema.

Lastly, like previous authors, we correlated MV or CSA with ASMM, but we have to keep in mind that these measurements do not represent the same physical attributes. Whereas one technique characterizes a volume through ultrasound, the other one derives total appendicular muscle mass through impedance analysis. If we consider sarcopenia as a disease, the most relevant points of comparison should be the consequences (i.e., falls, loss of mobility or death).

## 5 Conclusion

This study was the first to use freehand 3D‐US in a geriatric setting and develop a model to predict ASMM with ultrasound measurements in very old hospitalized patients. MV measurements with 3D‐US were reliable and more concordant with appendicular muscle mass and strength than 2D parameters. In opposition to MT or CSA, MV is a unique measurement per muscle and is highly repeatable. Moreover, on the contrary to BIA, 3D‐US gives the opportunity to follow specific MV changes, which could be useful to personalize resistance‐exercise techniques in a rehabilitation context.

## Ethics Statement

The study design complied with the Ethical guidelines for authorship and publishing in the Journal of Cachexia, Sarcopenia and Muscle.

## Conflicts of Interest

The authors declare no conflicts of interest.

## Supporting information


**Table S1** Concordance and accuracy matrix of Ultrasound measurements with Muscle mass and other Sarcopenia parameters, for men
**Table S2**: Concordance and accuracy matrix of Ultrasound measurements with Muscle mass and other Sarcopenia parameters, for women
**Figure S1**: Gender‐colourized correlation plots of ultrasound measurements with ASMM
**Figure S2**: Gender‐colourized correlation plots of ultrasound measurements with grip strength
