# Supplementary material for: Concordance of Freehand 3D Ultrasound Muscle Measurements With Sarcopenia Parameters in a Geriatric Rehabilitation Ward
Source: J Cachexia Sarcopenia Muscle. 2024 Nov 22;16(1):e13648. doi: 10.1002/jcsm.13648 (PMC11670163; doi:10.1002/jcsm.13648)
Supplement: Supplementary file 1 — Table S1 Concordance and accuracy matrix of Ultrasound measurements with Muscle mass and other Sarcopenia parameters, for men Table S2: Concordance and accuracy matrix of Ultrasound measurements with Muscle mass and other Sarcopenia parameters, for women Figure S1: Gender‐colourized correlation plots of ultrasound measurements with ASMM Figure S2: Gender‐colourized correlation plots of ultrasound measurements with grip strength [file JCSM-16-e13648-s001.docx]

**Supplemental material**

**Supplemental tables**

Table S1: Concordance and accuracy matrix of Ultrasound measurements with Muscle mass and other Sarcopenia parameters, for men

|  | Tibialis anterior | | | Rectus femoris | | | Vastus Lateralis | | |
| --- | --- | --- | --- | --- | --- | --- | --- | --- | --- |
|  | Volume | CSA | MT | Volume | CSA | MT | Volume | CSA | MT |
| BIA ASMM | 0.21  (0.93) | 0.49*  (0.79) | 0.57*  (0.77) | 0.17  (0.89) | 0.09  (0.82) | 0.12  (0.68) | 0.31  (0.90) | 0.31*  (0.69) | 0.21  (0.64) |
|  | | | | | | | | | |
| DXA  ASMM | 0.16  (0.83) | -0.04  (0.81) | 0.21  (0.74) | 0.23  (0.77) | 0.06  (0.79) | 0.09  (0.67) | 0.51  (0.84) | 0.47  (0.80) | 0.55*  (0.81) |
| Grip strength | 0.30  (0.98) | 0.12  (0.91) | 0.28  (0.88) | 0.33  (1) | 0.02  (0.91) | 0.06  (0.87) | 0.22  (0.99) | 0.38  (0.88) | 0.25  (0.79) |
| MVIC dorsiflexion | 0.47*  (0.92) | 0.08  (1) | 0.15  (0.99) | 0.20  (0.94) | 0.18  (0.99) | 0.17  (0.98) | 0.16  (0.94) | 0.12  (0.98) | 0.19  (0.95 |
| Knee extension torque | 0.47*  (0.98) | 0.34  (0.98) | 0.50*  (0.97) | 0.27 (0.98) | -0.03 (0.98) | 0.04  (0.94) | 0.62*  (0.99) | 0.62*  (0.95) | 0.50*  (0.89) |
|  | | | | | | | | | |
| FTSS | 0.24  (0.46) | 0.28  (0.60) | 0.24  (0.58) | 0.05  (0.43) | 0.11  (0.60) | 0.15  (0.70) | 0.13  (0.38) | 0.15  (0.68) | 0.08  (0.78) |
| Gait Speed | -0.03 (0.65) | -0.39  (0.81) | -0.18  (0.83) | 0.12  (0.69) | 0.02  (0.82) | 0.01  (0.93) | 0.05  (0.66) | 0  (0.92) | 0.13  (0.96) |

Table S2: Concordance and accuracy matrix of Ultrasound measurements with Muscle mass and other Sarcopenia parameters, for women

|  | Tibialis anterior | | | Rectus femoris | | | Vastus Lateralis | | |
| --- | --- | --- | --- | --- | --- | --- | --- | --- | --- |
|  | Volume | CSA | MT | Volume | CSA | MT | Volume | CSA | MT |
| BIA ASMM | 0.66*  (0.99) | 0.12  (0.95) | 0.09  (0.93) | 0.43*  (0.95) | 0.36*  (0.96) | 0.15  (0.94) | 0.41*  (0.97) | 0.17  (0.94) | 0.11  (0.90) |
|  | | | | | | | | | |
| DXA  ASMM | 0.31  (0.85) | 0.06  (0.91) | -0.14  (0.75) | 0.47  (0.96) | 0.13  (0.95) | 0.04  (0.80) | 0.20  (0.91) | 0.05  (0.77) | -0.15  (0.60) |
| Grip strength | 0.15  (0.90) | -0.21  (0.78) | -0.24  (0.76) | 0.05  (0.98) | 0.04  (0.70) | 0.13  (0.81) | 0.01  (0.96) | -0.09  (0.82) | 0.05  (0.71) |
| MVIC dorsiflexion | 0.24  (0.96) | -0.19  (1) | -0.28  (1) | 0.17  (0.83) | 0.04  (0.76) | -0.02  (0.99) | 0.12  (0.89) | 0.05  (0.99) | 0.16  (0.99) |
| Knee extension torque | 0.11  (0.99) | -0.16  (0.96) | -0.24  (0.95) | 0.10  (0.97) | 0.23 (0.91) | 0.19  (0.95) | 0.21  (0.97) | 0.17  (0.99) | 0.24  (0.93) |
|  | | | | | | | | | |
| FTSS | -0.02  (0.97) | 0.12  (0.94) | -0.05  (0.92) | -0.25  (0.81) | -0.24  (1) | -0.21  (0.86) | -0.04  (0.82) | -0.04  (0.93) | 0.02  (0.79) |
| Gait Speed | 0  (0.85) | -0.17  (0.95) | -0.03  (0.95) | 0.05  (0.75) | -0.24  (0.86) | 0.37*  (0.97) | -0.06  (0.72) | -0.17  (0.93) | 0.06  (0.98) |

**Supplemental Figures**


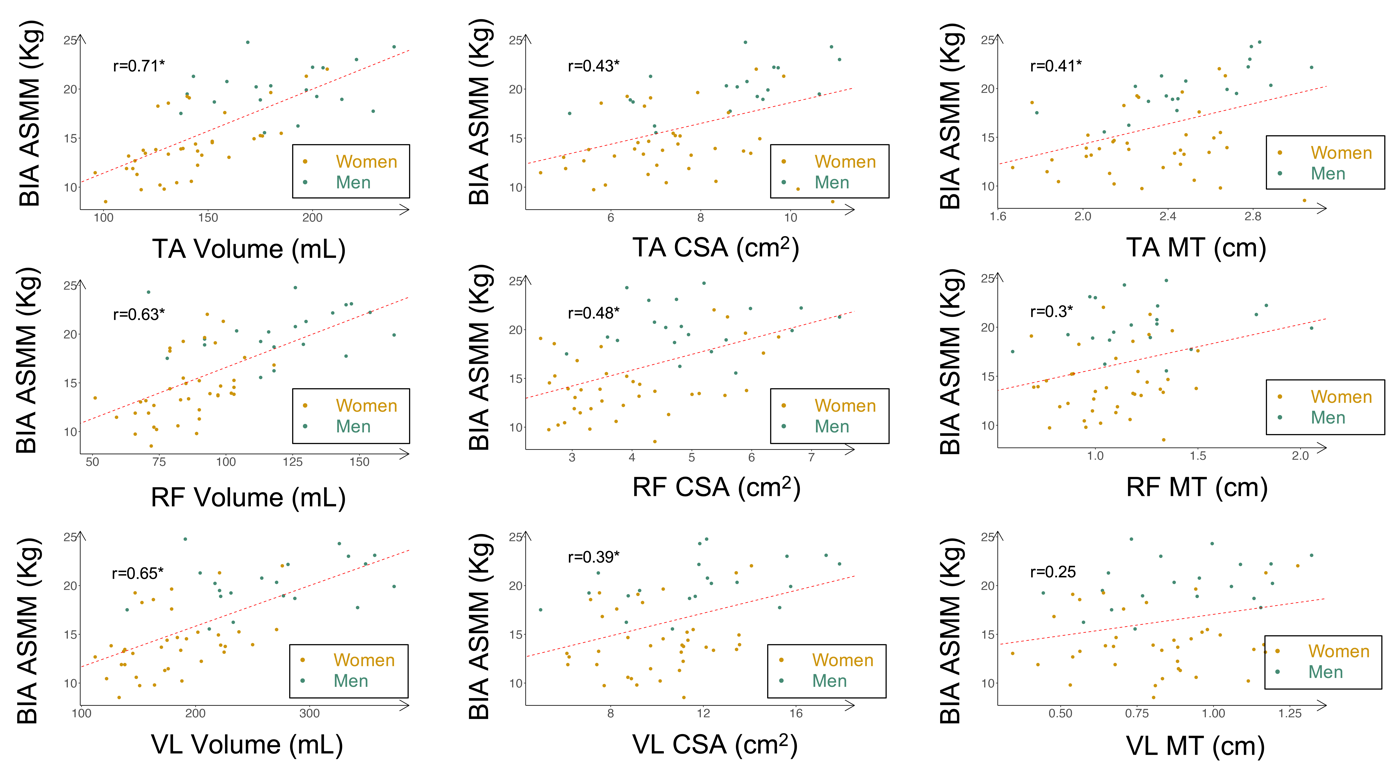


Fig. S1 Gender-colorized correlation plots of ultrasound measurements with ASMM


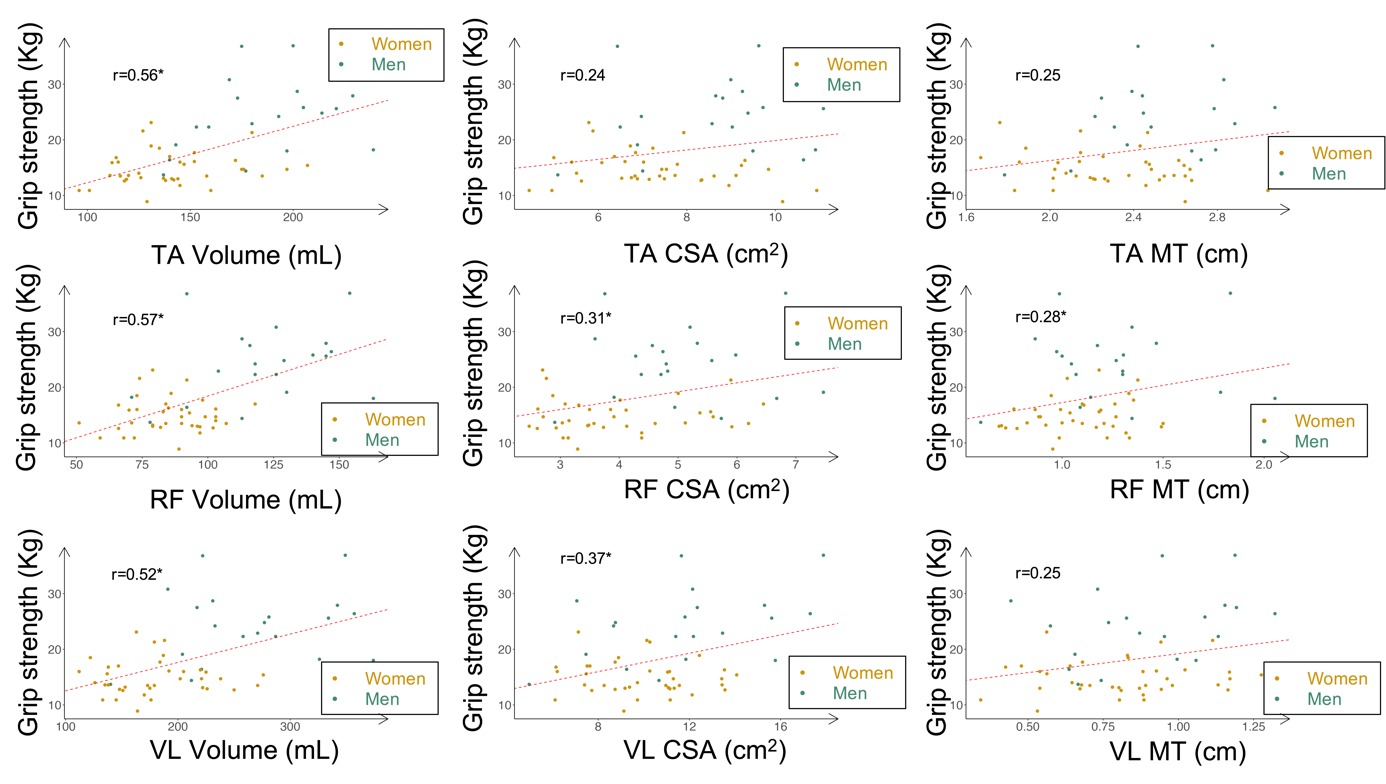


Fig. S2 Gender-colorized correlation plots of ultrasound measurements with grip strength
